# Circular RNA Expression in Oral Squamous Cell Carcinoma

**DOI:** 10.3389/fonc.2018.00398

**Published:** 2018-10-08

**Authors:** Yu-Fan Wang, Bo-Wen Li, Shuai Sun, Xiang Li, Wen Su, Zhi-Hong Wang, Feng Wang, Wei Zhang, Hong-Yu Yang

**Affiliations:** ^1^Department of Oral and Maxillofacial Surgery, Peking University Shenzhen Hospital, Shenzhen, China; ^2^Clinical School, Peking University Shenzhen Hospital, Anhui Medical University, Hefei, China; ^3^Biomedical Research Institute, Shenzhen Peking University-The Hong Kong University of Science and Technology Medical Center, Shenzhen, China

**Keywords:** Oral squamous cell carcinoma, circRNAs, noncoding RNA, oncogenesis, pathological differentiation

## Abstract

Circular RNA (circRNA) is a type of non-coding RNA molecule that affects the cellular regulatory network by sequestering microRNA (miRNA) like a sponge. This study was performed to identify differentially-expressed circRNA in oral squamous cell carcinomas (OSCCs). By high-throughput sequencing, microarray circRNA expression profiles were acquired from patients with OSCCs (*n* = 8) and controls (*n* = 8), which totaled 1921 existing circRNA molecules and 10021 novel circRNA molecules. Most of the circular RNA is from exons and distributed in the No. 1 and 2 chromosomes. Eight up-regulated and down-regulated circRNA molecules were identified as differentially-expressed in OSCCs. Among this, the expression of circ_000334, circ_006740, and circ_006371 are significantly down-regulated in 42 pairs of samples, which means that these circRNA molecules might be implicated in oncogenesis and development of OSCCs.

## Introduction

Head and neck squamous cell carcinoma (HNSCC), which affects 600,000 new patients each year, is the sixth most common malignancy worldwide(1, 2). It occurs in the oral cavity, oropharynx, larynx, and hypopharynx and accounts for over 90% of head and neck cancers in China (3). The current preferred therapy for HNSCC usually requires multimodal therapy combining surgery, radiotherapy, chemotherapy, and biotherapy. Surgery and radiotherapy are the major treatments. Recent studies have shown that preoperative chemotherapy does not improve overall survival and preoperative radiotherapy increases the risk of complications (4). The 5-years survival rate is 50%, and the long-term survival rate has only marginally improved (5–7). One important reason is that the mechanism of squamous cell carcinoma is unclear.

Recent advances in whole-exome sequencing and the discovery of non-coding RNA (ncRNA) have provided new insights into the molecular pathogenesis of HNSCC. A growing number of studies have shown the molecular-pathological participation of ncRNA, such as microRNA and lncRNA, in HNSCC formation and metastasis (8, 9). miRNA plays an important role in HNSCC molecular pathogenesis, such as tumor initiation and aggressiveness, regulation of cancer cell development and progression, mediation of AKT/mTOR signaling, and so on (10–12). LncRNA expression and function influences esophageal squamous cell carcinoma development. Additionally, lncRNA-based therapeutics show great potential in esophageal squamous cell carcinoma treatment. In 2016, Hongyu Yang et al. showed that increased expression of MALTA1 and UCA1 play an important role in metastasis of oral squamous cell carcinoma (OSCC) (13–15).

Circular RNA (circRNA) is a novel non-protein coding RNA. It is a form of RNA whose head 3′ and tail 5′ ends covalently bond together to result in a circular form (16). Although it was first identified in RNA viruses 40 years ago, its role in gene regulation and cancer formation have only recently been discovered (17). CircRNA is circular in shape, and function of circRNA is caused by the structure of miRNA response elements. By binding miRNA with miRNA response elements, circRNA serves as a miRNA sponge to regulate miRNA activity (18). Further functional roles, such as binding and sequestering RNA-binding proteins and building RNA-protein complexes, have also been discovered (19, 20). Recently, similar to miRNA and lncRNA in HNSCCs, dysregulation of circRNA expression has been identified in malignant tumor cells (breast, cervical, gastric, and oral cancer) (21, 22). circRNA also participates in regulating cancer-related pathways and as a proliferative factor and prognostic marker in gastric cancer (23). However, the role and mechanisms of circRNA underlying OSCC development and progression are unclear. This study was initiated to identify the role of circRNA in OSCCs by high-throughput sequencing.

## Materials and methods

### Patients and tissue samples

Eight pairs of snap-frozen oral squamous cell carcinoma tissue and adjacent normal tissue were acquired from OSCC patients by operation for circRNA High-throughput Deep Sequencing. Subsequently, a total of 42 pair samples including the samples for High-throughput Sequencing analysis were used for the circRNA validation by reverse transcriptase quantitative PCR(RT-qPCR). All experimental patient samples did not undergo other treatments before the operation and all oral squamous cell carcinoma tissue were confirmed by strict pathologic examination. Clinical and pathologic characteristics of patients were based on the WHO classifcation and UICC TNM classification. We obtained written informed consent from all patients and the study was approved by the Medical Ethics Committee of the Peking University ShenZhen Hospital. I confirmed that all methods were performed in accordance with the relevant guidelines and regulations.

### RNA extraction

After being obtained from surgical specimens, Samples (eight OSCC and matched para-carcinoma tissue) were immediately frozen using liquid nitrogen. Total RNAs were extracted from frozen tissues using TRIzol (Life Technologies, Scotland, UK) according to the manufacturer's instructions. RNA qualities were evaluated by Nano Drop ND-1000 spectrophotometer (Nano Drop Termo, Wilmington, DE).

### Library construction and sequencing

After extracted, total RNAs were treated with RNase R to degrade the linear RNAs, and purified using RNeasy MinElute Clean up Kit (Qiagen). Next, strand-specific library was constructed using VAHTS Total RNA-seq (H/M/R) Library Prep Kit for Illumina following the manufacturer's protocol. Briefly, ribosomes RNAs were removed to retain circRNAs. The enriched circRNAs were fragmented into short fragments by using fragmentation buffer and reverse transcripted into cDNA with random primers. Second strand cDNA were synthesized by DNA polymerase I, RNase H, dNTP (dUTP instead of dTTP) and buffer. Next, the cDNA fragments were purified with VAHTSTM DNA Clean Beads, end repaired, poly (A) added, and ligated to Illumina sequencing adapters. Then UNG (Uracil-N-Glycosylase) was used to digest the second-strand cDNA. The digested products were purified with VAHTSTM DNA Clean Beads; PCR amplified, and sequenced using Illumina HiSeqTM 2500 by Gene Denovo Biotechnology Co. (Guangzhou, China).

### Quantification of circRNA abundance

Reads obtained from the sequencing machines included raw reads containing adapters or low quality. To quantify high quality circRNAs, the back-spliced junction reads were scaled to RPM (Reads Per Million mapped reads). The RPM method is able to eliminate the influence of different sequencing data amount on the calculation of circRNA expression. Therefore, the calculated expression can be directly used for comparing the differential expression among samples.

### Data analysis

To identify differentially expressed circRNAs across samples or groups, the edgeR package (https://www.bioconductor.org/) was used. We identified circRNAs with a fold change ≥ 2 and a *P*-value < 0.05 in a comparison between samples or groups as significant differentially expressed circRNAs, then circRNAs were blasted against the circBase (23) for annotation. Those cannot be annotated were defined as novel circRNAs.

### Integrated analysis of circRNAs-miRNAs-mRNAs

For circRNAs (24) that have been annotated in circBase, the target relationship with miRNAs can be predicted by StarBase (v2.0) and three softwares Mireap, Miranda (v3.3a), and TargetScan (Version: 7.0) were used to predict targets for novel circRNAs. In order to predict the association of mRNAs with circRNAs and miRNAs, miRTarBase (v6.1) was used to predict mRNAs targeted by miRNAs sponge. The resulting correlation of circRNAs-miRNAs-mRNAs can be visualized by Cytoscape 3.01.

### Quantitative real-time PCR

Total RNA isolated from 42 samples was reversely transcribed into cDNA using the Prime Script RT Master Mix (Takara, Cat. #RR047A). Quantitative RT-PCR was conducted in Roche Applied Science LightCycler® 96 Real-time PCR systems (Roche Diagnostics, Indianapolis, Indiana, USA) in accordance to the manufacturer's instructions. A comparative cycle threshold (Ct) method was used to analyze the circRNA expression level. We used β-actin as an internal control and tested circRNA_006740 expression levels by qRT-PCR.

### Statistics

Significant different data from the High-throughput Sequencing were analyzed by *t-*test between different groups. CircRNAs having fold changes ≥2 and *p*-values ≤ 0.05 were selected as the significantly differentially expressions. PCR data were presented as mean ± standard error of the mean (SEM). Significant differences were determined by Student *t*-test and Spearman correlation test.

## Results

### Profile of differentially-expressed circRNA in OSCC patients

**Figure 1:**
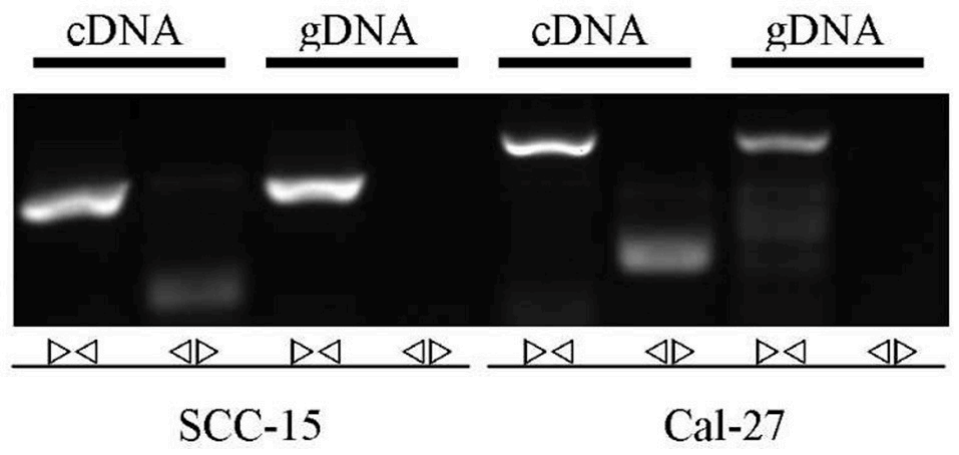
Divergent primers detect circular RNAs in cDNA but not genomic DNA (gDNA).

**Figure 2:**
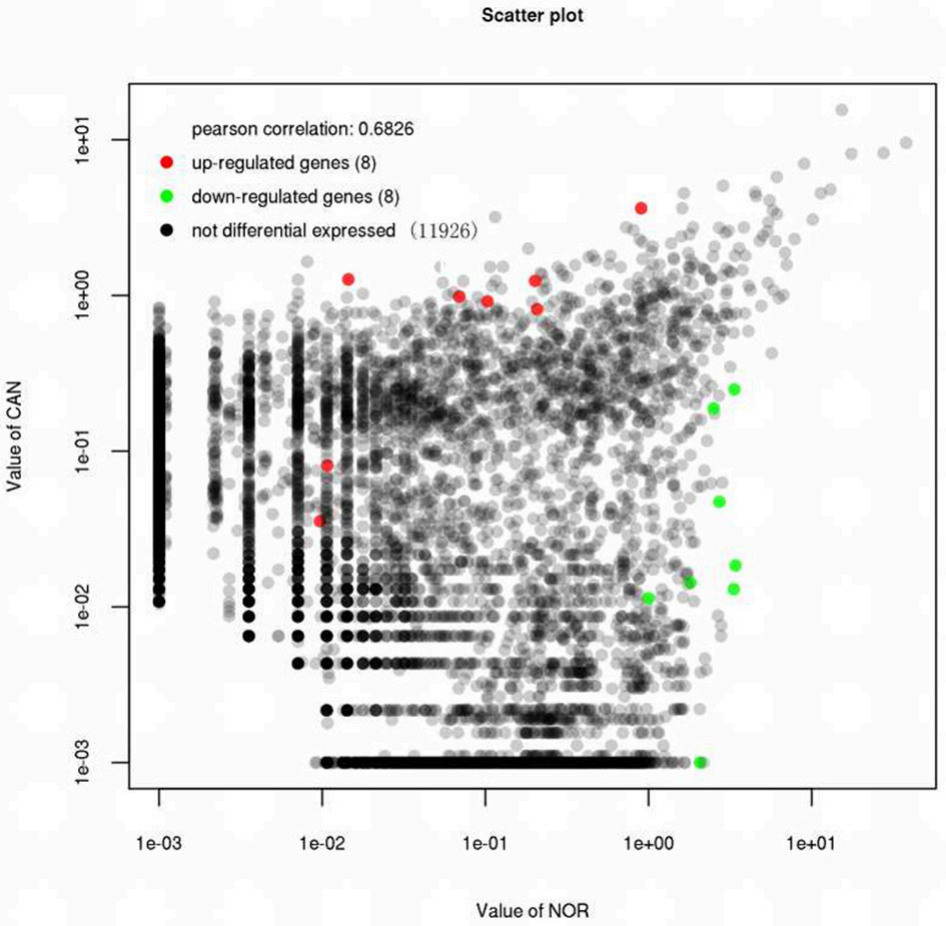
The expression of circRNAs in the eight pairs of OSCC tissue and adjacent normal tissue through the High-throughput Sequencing. 8 circRNAs were up-regulated (red spot) and 8 circRNAs (green spot) were down-regulated in the samples.

**Figure 3:**
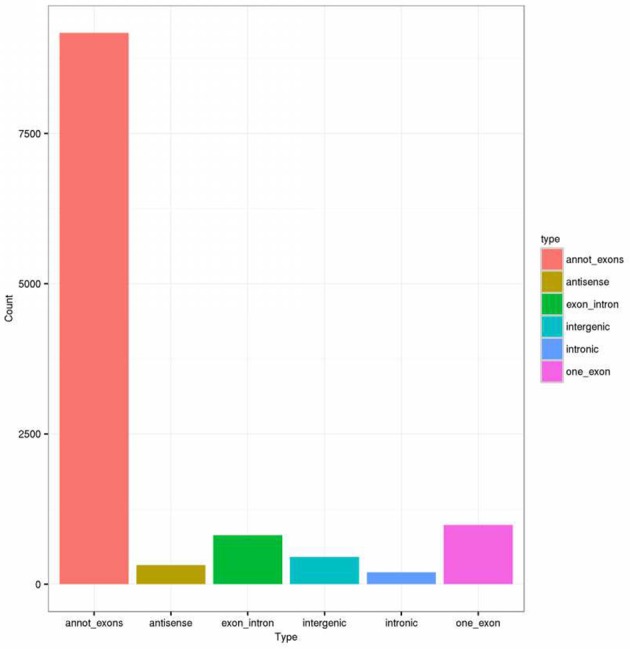
The bar diagram shows the circRNA category. Most of differentially expressed circRNAs originate from the exons. Some are from introns, while, a few are other sources.

**Figure 4:**
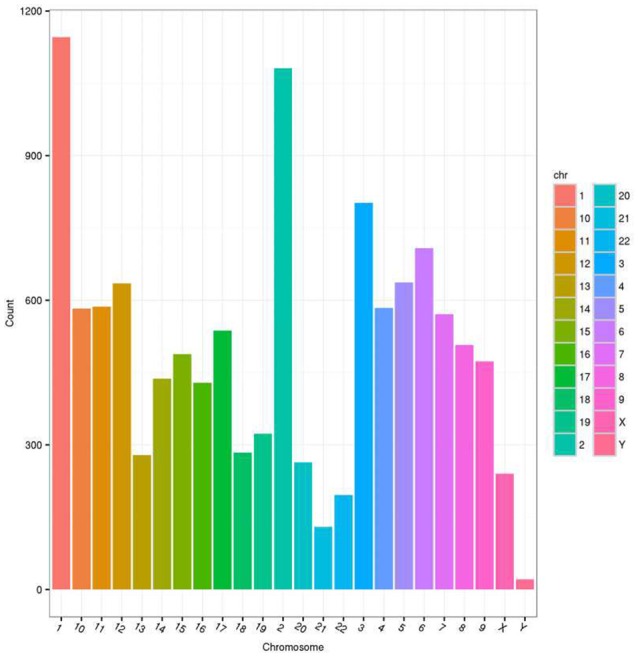
The distribution of differentially expressed circRNAs in human chromosomes.

**Figure 5:**
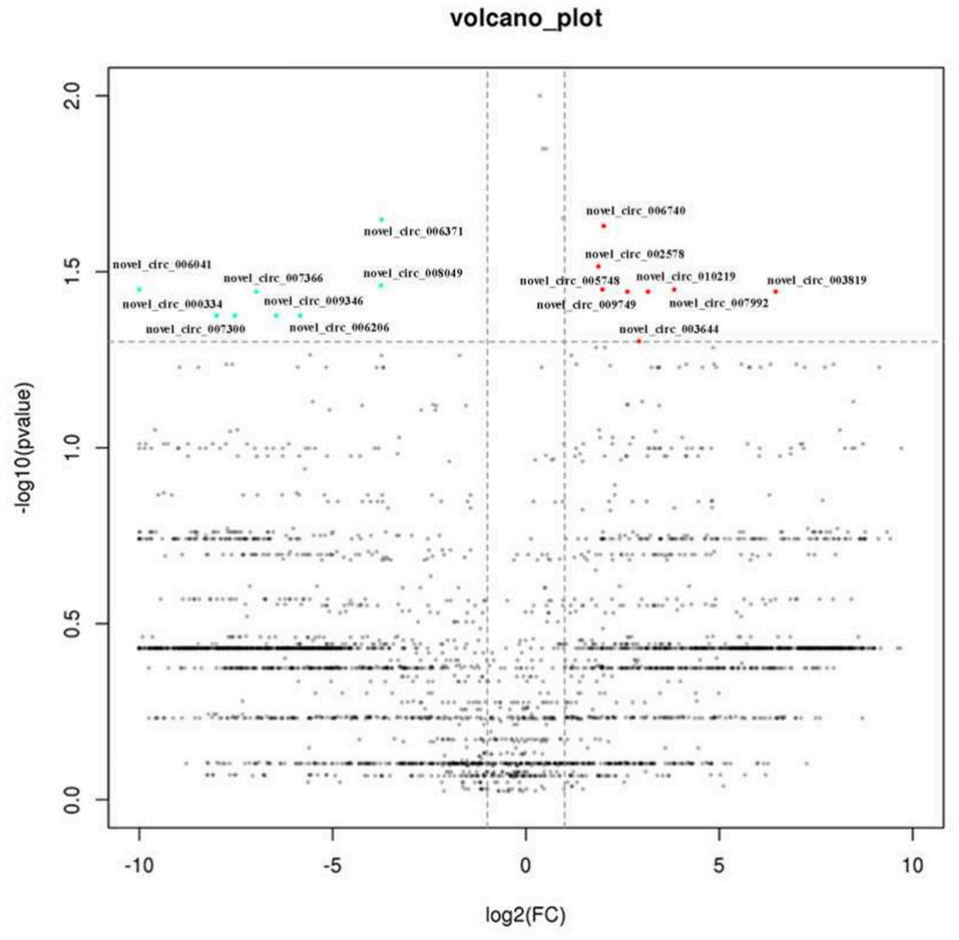
Volcano plots of the differential expressed circRNAS. The vertical lines correspond to 2.0 fold (log2 scaled) up and down, respectively, and the horizontal line represents a *P*-value of 0.05 (–log10 scaled). The red and green spots in plot represent the differentially expressed circRNAs with statistical signifcance.

**Figure 6:**
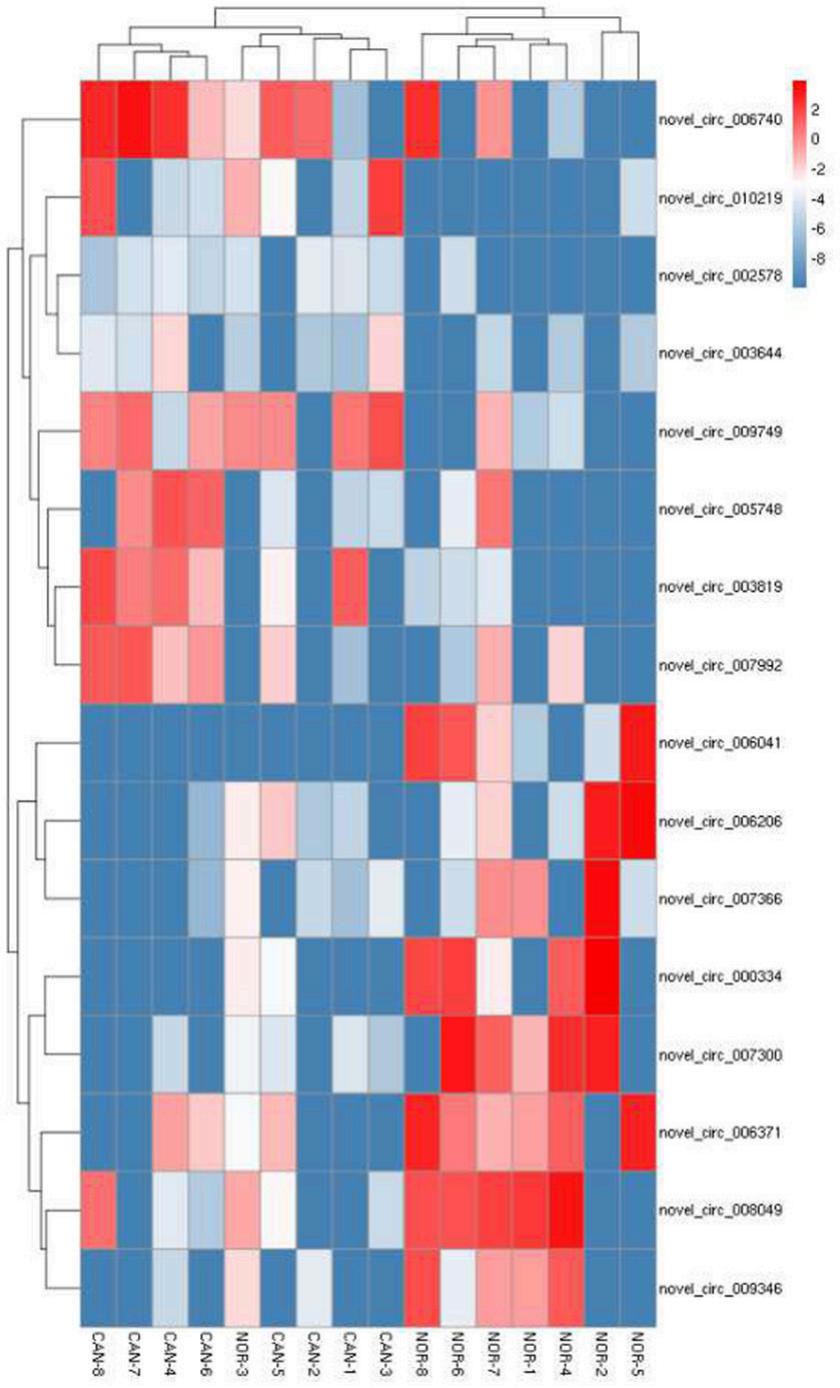
Heat map and hierarchical clustering showing expression values of the most up and down regulated circRNAs.

First, we designed two sets of primers for circRNA. The first set contained a divergent primer that amplifies only the circular form. The second set of primers contained an opposite-directed primer to detect the linear forms. PCR results indicate that the circular form was amplified using the divergent primers (Figure 1). PCR assays using cDNA and genomic DNA as templates did not produce amplifications with the divergent primers. We found a large amount of circRNA in the eight pairs of OSCC and adjacent normal tissue through high-throughput sequencing. A total of 11942 circRNA targets, including 1921 existing circRNA molecules and 10021 novel circRNA molecules, were detected in eight pairs of samples, then the scatter plot of circRNA expression profile was used to assess the variations between the two groups (Figure 2). Most circRNA is transcribed from protein coding exons. Some are from introns and are intragenic and antisense (Figure 3). The distribution of circRNA on human chromosomes is mostly concentrated on the first and second chromosomes (Figure 4). Differentially-expressed circRNA having statistical significance was confirmed with fold change filtering between two groups and was displayed through volcano plot filtering (Figure 5). Consequently, 16 circRNA molecules were detected to cause dysregulation by statistical analysis, including fold changes ≥ 2.0, *P* < 0.05 and FDR < 0.05 (Figure 6). Among these, eight circRNA molecules were up-regulated and eight were down-regulated in the samples (Table 1).

**Table 1:** 16 differently expressed circRNAs in the oral squamous cell carcinoma.

**circRNA**	**Fold change**	***P*-value**	**Type**	**chr**	**GeneSymbol**
hsa_circ_0008202	−8.00076	0.042153	Exons	1	SPATA6
hsa_circ_0004491	−3.73247	0.022494	Exons	2	ORC4
hsa_circ_0008309	2.010811	0.023438	Exons	2	CUL3
novel_circ_006041	−11.0136	0.035522	Exons	2	FANCL
novel_circ_007300	−7.53215	0.042153	One_exon	22	MB
novel_circ_007366	−6.97964	0.036032	Exons	22	FBLN1
novel_circ_009346	−6.46152	0.042153	Exons	5	EBF1
novel_circ_006206	−5.84394	0.042153	Exons	2	RMND5A
novel_circ_008049	−3.74847	0.034611	Exons	3	PHC3
novel_circ_002578	1.872629	0.030545	Antisense	12	KRT6C
novel_circ_005748	1.977281	0.035522	Exons	2	MBOAT2
novel_circ_009749	2.620575	0.036032	Exons	6	PHIP
novel_circ_003644	2.918808	0.049827	Intergenic	14	NA
novel_circ_007992	3.151974	0.036032	Exons	3	RNF13
novel_circ_010219	3.832216	0.035522	Exons	7	FAM126A
novel_circ_003819	6.455806	0.036032	Exons	15	SPPL2A

### CircRNA molecules' gene symbols by GO analysis and pathway analysis

**Figure 7:**
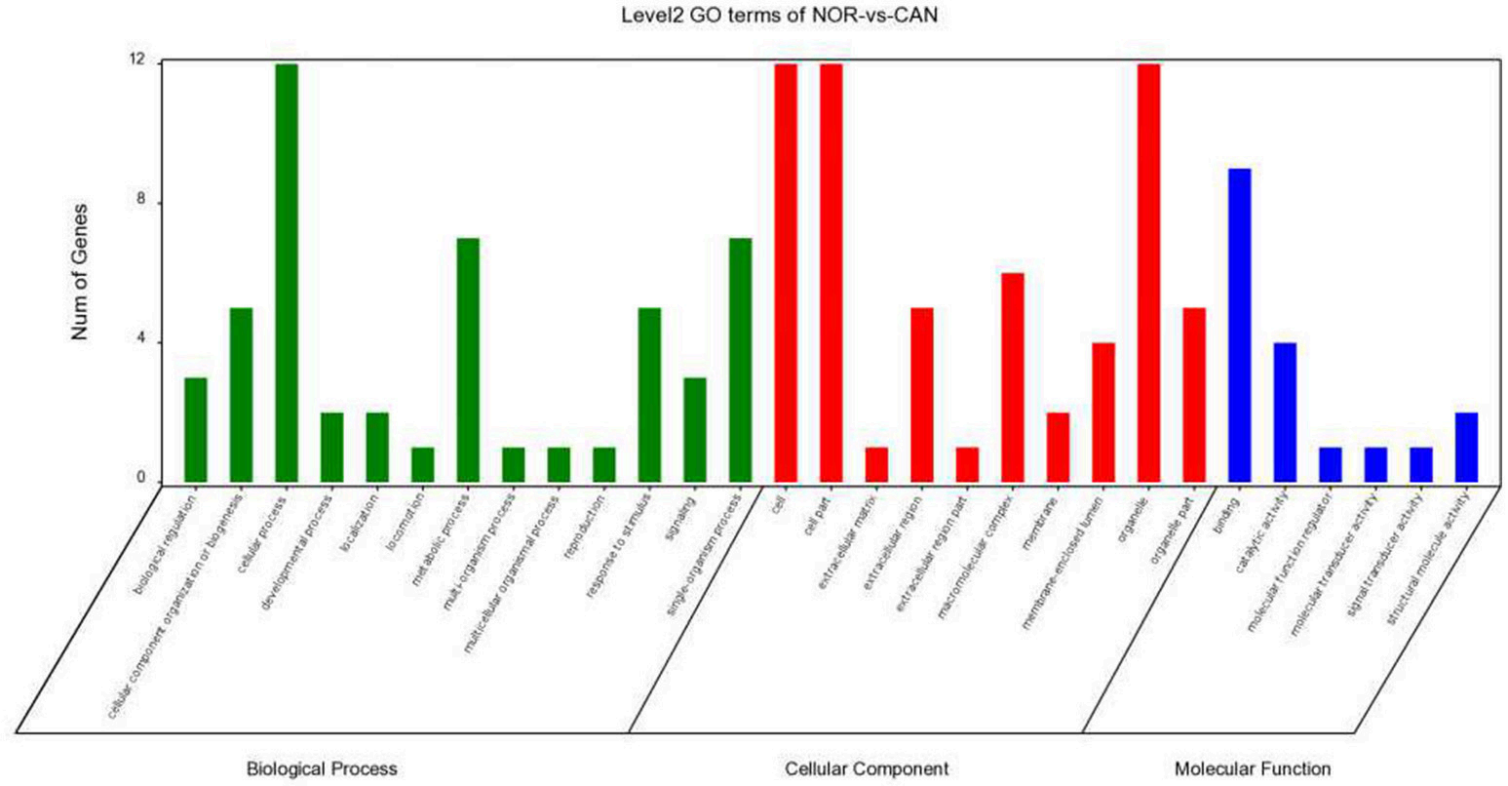
GO enrichment analysis for circRNAs gene symbols.

**Figure 8:**
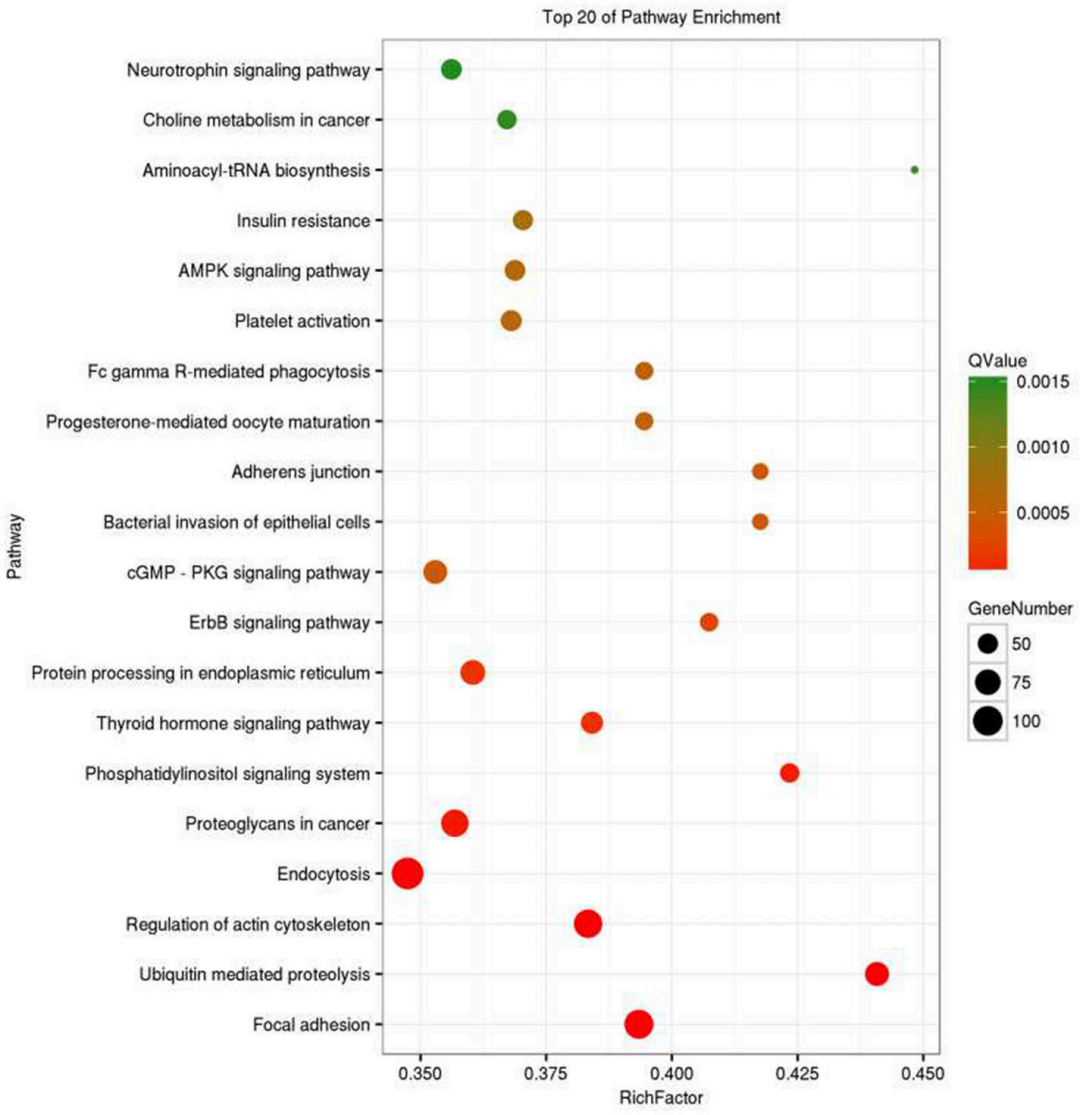
Top 20 of pathway enrichment analysis for circRNAs gene symbols.

Recent research has been reported that circRNA is associated with the parental genes (25–27); therefore, we analyzed circRNA gene symbols using GO analysis and pathway analysis and speculated circRNA potential functions. We found that the most significant enriched GO term in the biological process was cellular process (GO: 0009987), the most significant enriched GO term in the cellular component was cell (GO: 0005623), and the most significant enriched GO term in the molecular function was binding (GO: 0005488) (Figure 7). Pathway analysis indicated that the top 20 pathways might be involved in the progression of OSCC Figure 8. Among these pathways, the gene category involving ErbB and AMPK signaling pathway has been reported to be related to the progression of OSCC (28, 29).

### Prediction for the circRNA/miRNA interaction

**Figure 9:**
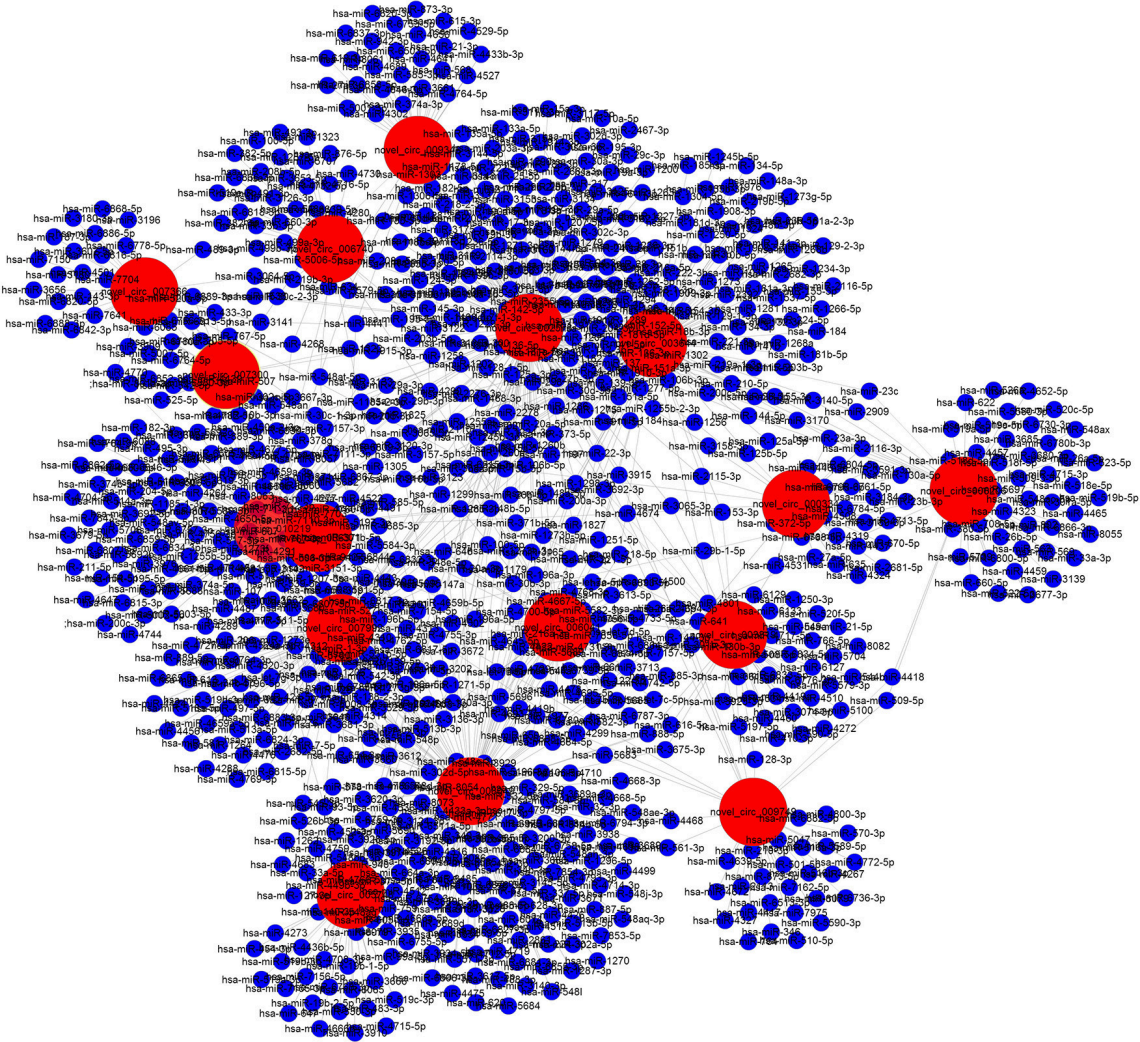
The dysregulated circRNA/miRNA network analysis.

**Figure 10:**
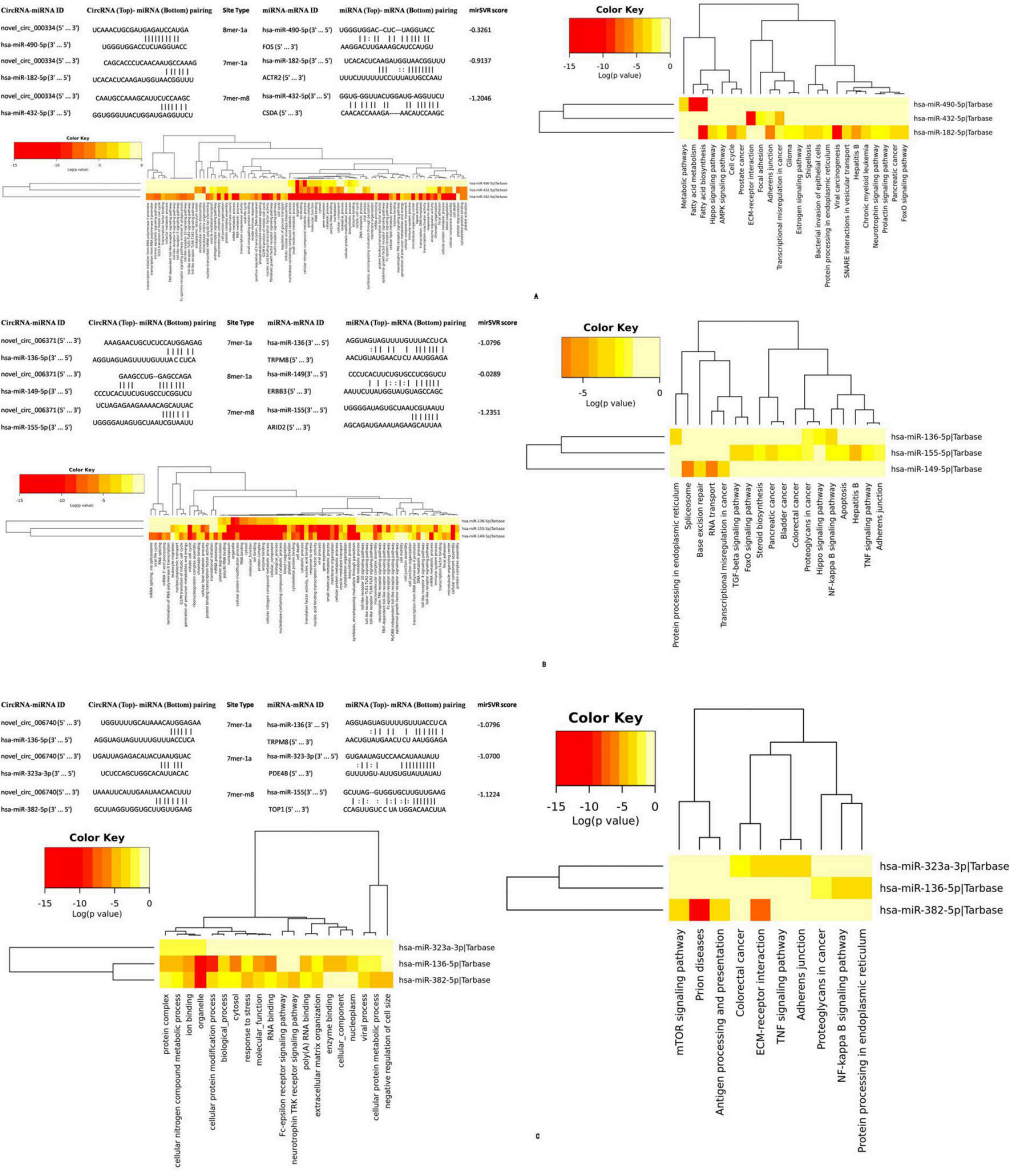
The role of miRNA targeted with circ_000334 **(A)**, circ_006371 **(B)**, and circ_006740 **(C)** by the StarBase and TargetScan databases.

To explore the molecular mechanism and functions of circRNA, we investigated potential miRNA binding with circRNA. For circRNA that has been annotated in circBase, the target relationship with miRNA can be predicted by StarBase. Novel circRNA was predicted using three programs: Mireap, Miranda, and TargetScan (Figures 9, 10).

### Validation of significant different circRNA and clinical characteristics

**Figure 11:**
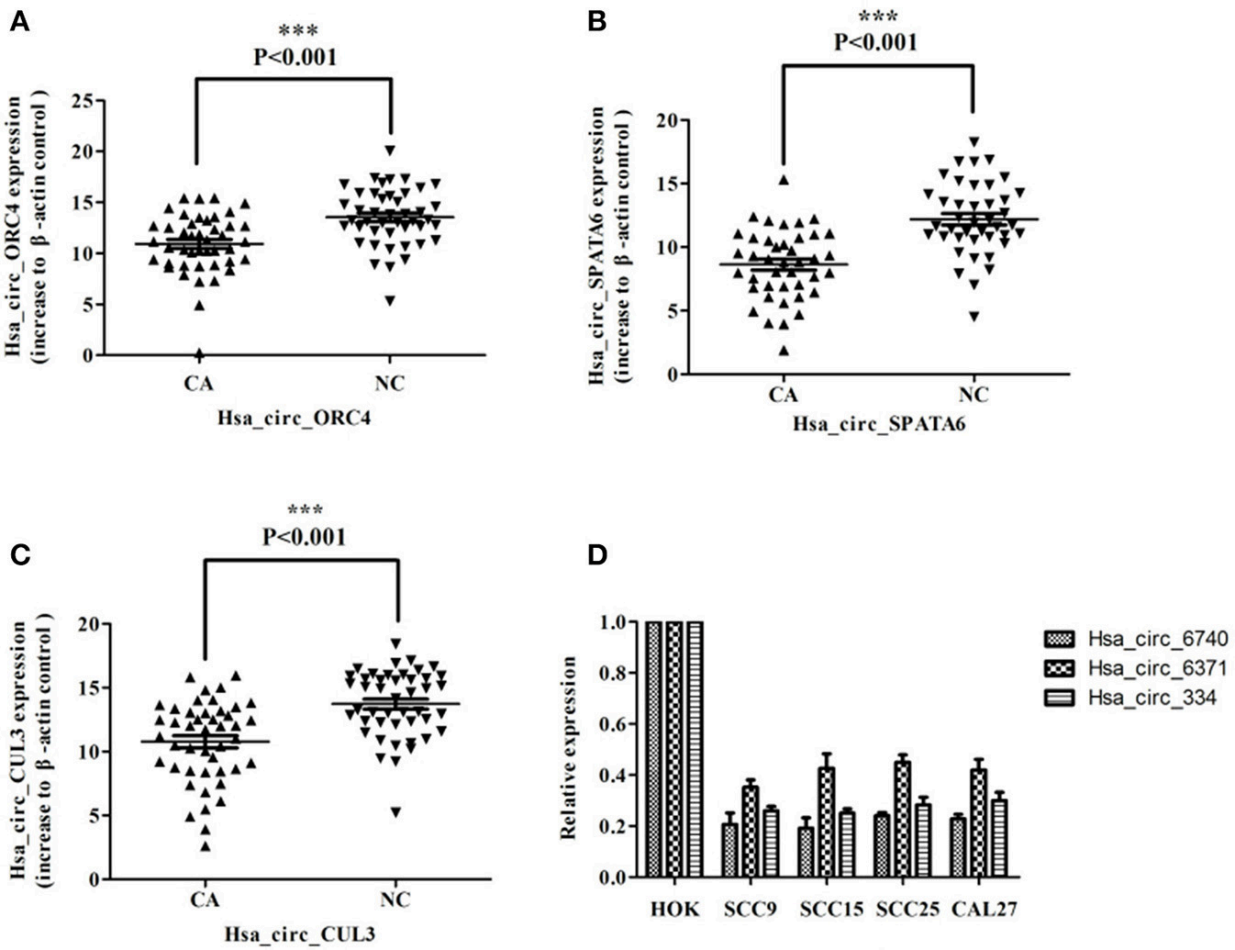
Analysis of hsa_circ_ORC4, hsa_circ_SPATA6 and hsa_circ_CUL3 in OSCC tissues and cell lines. **(A)** The levels of hsa_circ_ORC4 in OSCC tissues are significantly higher than those in normal tissues. **(B)** The levels of hsa_circ_SPATA6 in OSCC tissues are significantly higher than those in normal tissues. **(C)** The levels of hsa_circ_CUL3 in OSCC tissues are significantly higher than those in normal tissues. **(D)** The expression of hsa_circ_ORC4, hsa_circ_ORC4 and hsa_circ_CUL3 in OSCC cell lines (SCC9, SCC15, SCC25, CAL27) compared with HOK (normal oral keratinocyte cell line).

We further confirmed the high-throughput sequencing results by quantitative real-time PCR (qRT-PCR) in 42 pairs of samples including the tissues for high-throughput sequencing analysis. The results showed that three circRNA molecules (circRNA_000334, circRNA_006740, and circRNA_6371) were significantly down-regulated expression in carcinoma tissues and OSCC cell lines (Figure 11). circRNA_000334, circRNA_006740 were related to pathological differentiation Tables 2–4.

**Table 2:** Correlation between hsa_circ_006740 expression and clinicopathological characteristics in 42 oral squamous cell carcinoma patients.

**Items**	**Total cases (*n* = 42)**	***P*-value**
**Gender**		0.87
Male	31	
Female	11	
**Age (years)**		0.80
Range(median)	29–78(54)	
<60	27(46.185)	
≥60	14(66.348)	
**Site**		0.28
Tongue	23	
Buccal	7	
Gingiva	5	
Floormouth	7	
**Clinical stage**		0.29
0+I + II	17	
III + IV	25	
**Pathological differentiation**		0.05^*^
Well	18	
Moderately	18	
Poorly	6	
**LNM**		0.31
Yes	16	
No	26	

LNM, Lymph Node Metastasis.

* *P < 0.05*.

**Table 3:** Correlation between hsa_circ_006371 expression and clinicopathological characteristics in 42 oral squamous cell carcinoma patients.

**Items**	**Total cases (*n* = 42)**	***P*-value**
**Gender**		0.85
Male	31	
Female	11	
**Age (years)**		0.24
Range(median)	29-78(54)	
<60	27(46.185)	
≥60	14(66.348)	
**Site**		0.80
Tongue	23	
Buccal	7	
Gingiva	5	
Floormouth	7	
**Clinical stage**		0.46
0+I + II	17	
III + IV	25	
**Pathological differentiation**		0.95
Well	18	
Moderately	18	
Poorly	6	
**LNM**		0.77
Yes	16	
No	26	

*LNM, Lymph Node Metastasis*.

**Table 4:** Correlation between hsa_circ_000334 expression and clinicopathological characteristics in 42 oral squamous cell carcinoma patients.

**Items**	**Total cases (*n* = 42)**	***P*-value**
**Gender**		0.86
Male	31	
Female	11	
**Age (years)**		0.21
Range(median)	29-78(54)	
<60	27(46.185)	
≥60	14(66.348)	
**Site**		0.87
Tongue	23	
Buccal	7	
Gingiva	5	
Floormouth	7	
**Clinical stage**		0.34
0+I + II	17	
III + IV	25	
**Pathological differentiation**		0.039^*^
Well	18	
Moderately	18	
Poorly	6	
**LNM**		0.55
Yes	16	
No	26	

LNM, Lymph Node Metastasis.

* *P < 0.05*.

## Discussion

CircRNA is a new large class of ncRNA with the function of acting as a miRNA sponge. It was first found in RNA viruses in the 1970s. Unlike linear RNA, circRNA forms covalently closed loop structures and possesses no polyA tail (30). It is more stable than linear RNA and resistant to the activity of RNase, which is an exonuclease enzyme that degrades linear RNA molecules. The function of circRNA has rarely been studied using RNA sequencing technologies. circRNA is implicated in various diseases including cancer (31). In this research, we prove the role of circRNA in OSCCs.

We found that the expression of circular RNA is abundant in eight pairs of samples, including 1921 existing circRNA molecules and 10021 novel circRNA molecules (Figure 2). In our research, most of the circular RNA is from exons. Exonci circRNA is generated by a process called back-slicing, an out-of-order arrangement of exons (32). Intronic circRNA, which is derived from intron, is a stable circRNA with a 2′-5′ linkage. Most circular RNA involved in cellular function derived from exon (33). Moreover, circular RNA is mainly distributed in the No. 1 and 2 chromosomes. In these chromosomes, the significance of circRNA in OSCC was realized compared with normal tissue. Furthermore, many researchers have already associated dysregulation of circRNA in different malignant tumors. Eight up-regulated and down-regulated circRNA molecules were identified in this study, which means that circRNA might be implicated in the oncogenesis and development of OSCC.

circ_000334, circ_006740, and circ_006371 are spliced from spermatogenesis-associated protein 6 (SPATA6), CUL3, and SLC38A1, which play an important role in tumor proliferation, migration, and apoptosis. SPATA6 is involved in sperm formation and development of testicular cancer (28). CUL3 is one of the most important members of the Ubiquitin E3 enzyme family. Gene Keap1, KLHL20, and SPOP, which participate in the development and prognosis of tumors, can regulate the ubiquitination of CUL3 (34). SLC38A1 is one amino acid transporter that is involved in the regulation of cell metabolism, energy supply, and malignant change. The high expression of SLC38A1 was detected in gastric cancer, and was related with tumor differentiation degree, TNM staging, lymph node metastasis, and prognosis (35). Furthermore, the expressions of circ_000334, circ_006740, and circ_006371 are significantly down-regulated in the OSCCs. We also found that the downregulation of circ_000334 and circ_006740 was related to pathological differentiation. Based on this, we think that circRNA may participate in development and prognosis of OSCCs.

According to bioinformatics technology, we explore the function of the source gene in biological processes and molecular functions. We found that membrane-enclosed lumen affects the development of OSCCs (33). Moreover, ErbB and AMPK pathway has been reported to participate in the development and prognosis of OSCCs (28, 29). The literature reports that circular RNA has the function of acting as a miRNA sponge by regulating the miRNA to influence tumor development. Therefore, using the StarBase and TargetScan databases, we predict a relationship between the circular RNA and microRNA. We found that most circular RNA contains one or more binding sites for microRNA. Figure 11 shows the role of microRNA targeted with circ_000334, circ_006740, and circ_006371. Each of these proves that circRNA plays an important role in OSCC.

In conclusion, we found that circular RNA was significantly expressed in OSCCs compared with normal tissues. circRNA plays an important role in development of OSCCs and regulates cancer-related pathways. This study will help researchers to elucidate the mechanism of oral squamous carcinoma development and provide new clinical diagnostic markers and therapeutic target.

## Ethics statement

This study was carried out in accordance with the recommendations of medical ethics committee of Peking Univerisity Shenzhen Hospital with written informed consent from all subjects. All subjects gave written informed consent in accordance with the Declaration of Helsinki. The protocol was approved by the medical ethics committee of Peking Univerisity Shenzhen Hospital.

## Author contributions

Y-FW and B-WL: Design experiments. SS, XL and WS: Analysis of data. Z-HW and FW: Drafting the work. WZ and H-YY: Final approval of the version to be published.

### Conflict of interest statement

The authors declare that the research was conducted in the absence of any commercial or financial relationships that could be construed as a potential conflict of interest.
